# Comparison and Regulation of Neuronal Synchronization for Various STDP Rules

**DOI:** 10.1155/2009/704075

**Published:** 2009-07-16

**Authors:** Yanhua Ruan, Gang Zhao

**Affiliations:** Institute of Complex Bio-dynamics, Jiangxi Blue Sky University, Nanchang, Jiangxi 330098, China

## Abstract

We discuss effects of various experimentally supported STDP learning rules on frequency synchronization of two unidirectional coupled neurons systematically. First, we show that synchronization windows for all STDP rules cannot be enhanced compared to constant connection under the same model. Then, we explore the influence of learning parameters on synchronization window and find optimal parameters that lead to the widest window. Our findings indicate that synchronization strongly depends on the specific shape and the parameters of the STDP update rules. Thus, we give some explanations by analyzing the synchronization mechanisms for various STDP rules finally.

## 1. Introduction

Synchronous activity is a basic characteristic in the brain. It exists in many regions of the brain, such as CA1 of the hippocampus [1], visual cortex [2] and cortical areas correlating with conscious perception [3]. It is known that synchronization is very important for information processing, such as predicting sensory input [4], and information codes [5]. Moreover, synchronous activity plays a crucial role in epileptic activity [6, 7], modulation of neurons about attention [8], memory and learning [9, 10], and cognitive functions [11].

Since the discovery of long term potentiation (LTP) and LTD (long term depression) [12–14], it has been debated how synaptic modifications are correlated to neuron activities. Spike timing-dependent plasticity (STDP) is a form of synaptic modification discovered relatively recently, which depends on the relative timing of pre- and post-synaptic action potentials at a millisecond time scale [15, 16]. Many experiments have proved the existence of STDP, such as in neocortical slices [17], hippocampus slice [18], hippocampal cell cultures [19], and tadpole rectum in vivo [20]. In addition, STDP provides powerful mechanisms for models of temporal pattern recognition [21], temporal sequence learning [22, 23], a continuous-time associative memory [24], coincidence detection [16, 25], navigation [26, 27] and direction selectivity [28].

The interaction among neurons relies much on synaptic modification in which STDP is the only one that greatly expands the capability of Hebbian learning to address temporally sensitive computational tasks. STDP in synchronization has attracted wide interests. For example, the result of learning-induced synchronization of a neural network at various developing stages using STDP rule is consistent with recent experimental observations [29]. Furthermore, the comparison of synchronization between discontinuous anti-STDP(dc-aSTDP, see Section 2) and constant connection has been investigated [30]. Following it, the continuous STDP(c-STDP, see Section 2) has also been studied [31] by the same authors. They suggest that a functional role of STDP might be enhancing synchronization. Motivated by their work, we systemically discuss the roles of four types of STDP rules(c-STDP, dc-STDP, dc-aSTDP and in-STDP, see Section 2) in frequency synchronization in the present paper, employing the same model [31] with only values of some parameters different, such as *A*_plus_, *t*_syn_, *V*_slope_, *g*_max_ (see Section 3).

We find, however, not all STDP rules facilitate synchronization. It encourages us to trace the reason. We then consider if the learning curves, which characterize the STDP rule, have certain effects on synchronization. Results indicate that synchronization strongly depends on the specific shape and the parameters of the STDP rule. However, the optimal synchronization ranges for dc-STDP and in-STDP, got from regulating learning parameters, are not wider than those for the corresponding strongest constant connection respectively. As a result, when we seek the reason, we discover that the synchronization mechanisms of above four STDP rules can be classified into two categories: (i) c-STDP and dc-aSTDP rules; (ii) dc-STDP and in-STDP rules. The synchronization mechanisms of the two categories are different. For c-STDP and dc-aSTDP rules, two neurons' synchronization either relies on the balancing out potentiation and depression during one cycle consistent with the perspective of Nowotny et al. or relies on the maximal synaptic conductance. However, for dc-STDP and in-STDP rules, the synchronization windows are completely provided by the respective maximal synaptic conductance. As regards this finding, we offer an intuitive explanation finally.

## 2. Models and Method

We consider two HH neurons with unidirectional activity-dependent excitatory or inhibitory synaptic coupling. Although such a configuration is too simple to find applications in brain information processing, it serves as a staring point for many model researches. The neurons are modeled with standard Na, K, and “leak” currents [32],

(1) $$\begin{array}{ll} C\frac{dV_{i}\left(t\right)}{dt} & =-g_{\text{Na}}\cdot m_{i}{\left(t\right)}^{3}\cdot h_{i}\left(t\right)\cdot \left(V_{i}\left(t\right)-E_{\text{Na}}\right)-g_{\text{K}}\cdot n_{i}{\left(t\right)}^{4}\\ & \;\cdot \left(V_{i}\left(t\right)-E_{\text{K}}\right)-g_{L}\cdot \left(V_{i}\left(t\right)-E_{L}\right)-I_{\text{syn}}\left(t\right)+I_{\text{stim}}, \end{array}$$

where *i* = 1, 2.

Each of the activation and inactivation variables *y*_*i*_(*t*) = {*n*_*i*_(*t*), *m*_*i*_(*t*), *h*_*i*_(*t*)}, *i* = 1, 2 satisfies first-order kinetics,

(2) $$\frac{dy_{i}\left(t\right)}{dt}=\alpha_{y}\left[V_{i}\left(t\right)\right]\left[1-y_{i}\left(t\right)\right]-\beta_{y}\left[V_{i}\left(t\right)\right]y_{i}\left(t\right),\;i=1,2.$$

The parameters in these equations are given in [31],

(3) $$\begin{array}{ll} \alpha_{n} & =\frac{0.032\left(-50-V\right)}{\exp\left(\left(-50-V\right)/5\right)-1},\\ \beta_{n} & =0.5\exp(\left(-55-V\right)40),\\ \alpha_{m} & =\frac{0.32\left(-52-V\right)}{\exp\left(\left(-50-V\right)/4\right)-1},\\ \beta_{m} & =\frac{0.28\left(25+V\right)}{\exp\left(\left(25+V\right)/5\right)-1},\\ \alpha_{h} & =0.128\exp\left(\frac{-48-V}{18}\right),\\ \beta_{h} & =\frac{4}{\exp\left(\left(-25-V\right)/5\right)+1}. \end{array}$$

*I*_stim_ is a constant input current forcing each neuron to spike with a constant, *I*_stim_-dependent period, labeled as *T*_1_ and *T*_2_. The postsynaptic neuron would show another firing period *T*_2_^1^, when it is driven by the synaptic current, which is dependent on the postsynaptic potential *V*_2_(*t*), the reversal potential *V*_rev_, the activation variable *S*(*t*) and its maximal conductance *g*(*t*),

(4) $$I_{\text{syn}}\left(t\right)=g\left(t\right)S\left(t\right)\left(V_{2}\left(t\right)-V_{\text{rev}}\right),$$

where

(5) $$\begin{array}{cc} \frac{dS\left(t\right)}{dt} & =\frac{S_{\infty }\left(V_{1}\left(t\right)\right)-S\left(t\right)}{t_{\text{syn}}\cdot 1-\left(S_{\infty }\left(V_{1}\left(t\right)\right)\right)},\\ S_{\infty }\left(V\right) & =\{\begin{array}{cc} \text{tanh}\;\left(\frac{V-V_{th}}{V_{\text{slope}}}\right), & \text{for}\;V>V_{th},\\ 0, & \text{otherwise}. \end{array} \end{array}$$

The time-dependent synaptic coupling strength *g*(*t*) nS is

(6) $$g\left(t\right)=\frac{g_{\max\;}}{2}\left(\text{tanh}\;\left(\frac{g_{\text{raw}}-g_{\text{mid}}}{g_{\text{slope}}}\right)+1\right).$$

Therefore *g*(*t*) always have values between 0 nS and *g*_max_. The bound imposed on *g*(*t*) is artificially set to avoid unrealistically high synaptic conductance and negative conductance. In order to obtain biologically plausible synaptic conductance, several methods have been employed to limit the synaptic strength in literature, such as a negative total integral [33], artificial bounds [34], and self-limitation [31]. Unless otherwise stated, we employ the self-limitation method which is characterized by a function “tanh” in our simulation.

*g*_raw_ is modified by STDP rules that are introduced in the next paragraph. The initial value of *g*_raw_ is 20 nS. The parameters of the model are

(7) $$\begin{array}{c} C=30\;\mu \text{F},\;\;g_{L}=1\;\mu \text{S},\;\;E_{L}=-64\;\text{mV},\\ g_{\text{Na}}=360\;\mu \text{S},\;\;E_{\text{Na}}=50\;\text{mV},\;\;g_{\text{K}}=70\;\mu \text{S},\\ E_{\text{K}}=-95\;\text{mV},\;\;V_{th}=-20\;\text{mV},\;\;t_{\text{syn}}=25\;\text{ms},\\ V_{\text{slope}}=15\;\text{mV},\;\;g_{\max\;}=25\;\text{nS},\;\;g_{\text{mid}}=\frac{1}{2}g_{\max\;},\\ g_{\text{slope}}=g_{\text{mid}},\;\;V_{\text{rev}}=20\;\text{mV}. \end{array}$$

**Figure 1:**
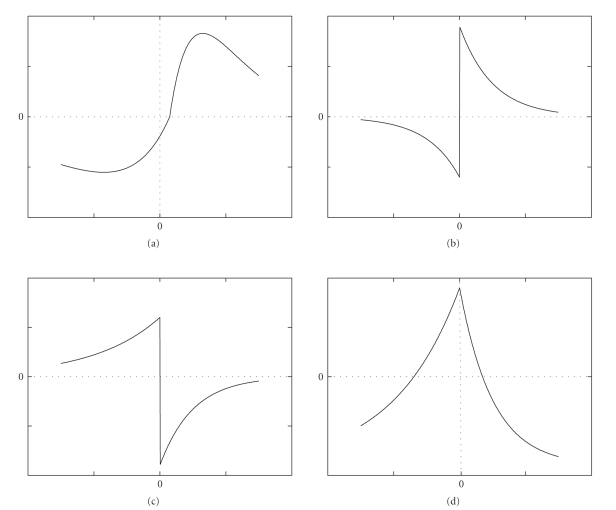
Different types of STDP curves are presented. (a) (c-STDP ) (b) discontinuous STDP(dc-STDP); (c) discontinuous anti-STDP(dc-aSTDP); (d) inhibitory STDP(in-STDP).

The time-dependent synaptic coupling strength *g*(*t*) is determined by the spike-timing of pre- and postsynaptic spikes. We consider four types of activity-dependent couplings that have been found in experiments: (1) an excitatory synapse with continuous STDP (c-STDP). There are two forms of c-STDP from two different experiments. One (Figure 1(a)) is from the recording of the neocortex-layer 5 Xenopus tectum hippocampus [31, 35], and the other is from the neocortex-layer 4 spiny stellates [36]. The latter form will not be considered here, because it introduces persistent decrease to synaptic strength that would result in none synchronization if two neurons have different inherent periods. (2) an excitatory synapse with discontinuous STDP (dc-STDP, Figure 1(b)) [17, 36]; (3) an excitatory synapse with discontinuous anti-STDP [36, 37] (dc-aSTDP, Figure 1(c)); (4) an inhibitory synapse with STDP (in-STDP, Figure 1(d)) [18, 19, 36].

Δ*g*_raw_ is a function of Δ*t* = *t*_postspike_ − *t*_prespike_, time difference between the times of postsynaptic and presynaptic spikes. The learning rules corresponding to Figures 1(a), 1(b), 1(c), 1(d)) are provided as follows

(8) $$\begin{array}{ll} \text{c-STDP:} & \\ \Delta g_{\text{raw}} & =\{\begin{array}{cc} A_{\text{plus}}\frac{\Delta t-\tau_{0}}{t_{\text{plus}}}\cdot e^{-\Delta t/t_{\text{plus}}}, & \Delta t>\tau_{0},\\ A_{\text{sub}}\frac{\Delta t-\tau_{0}}{t_{\text{sub}}}\cdot e^{\Delta t/t_{\text{sub}}}, & \Delta t\leq \tau_{0}, \end{array}\\ \text{dc-STDP:} & \\ \Delta g_{\text{raw}} & =\{\begin{array}{cc} A_{\text{plus}}\cdot e^{-\Delta t/t_{\text{plus}}}, & \Delta t>0,\\ -A_{\text{sub}}\cdot e^{\Delta t/t_{\text{sub}}}, & \Delta t\leq 0, \end{array}\\ \text{dc-aSTDP:} & \\ \Delta g_{\text{raw}} & =\{\begin{array}{cc} -A_{\text{plus}}\cdot e^{-\Delta t/t_{\text{plus}}}, & \Delta t>0,\\ A_{\text{sub}}\cdot e^{\Delta t/t_{\text{sub}}}, & \Delta t\leq 0, \end{array}\\ \text{in-STDP:} & \\ \Delta g_{\text{raw}} & =\{\begin{array}{cc} A_{\text{plus}}\cdot \left(e^{-\Delta t/t_{\text{plus}}}-0.5\right), & \Delta t>0,\\ A_{\text{sub}}\cdot \left(e^{\Delta t/t_{\text{sub}}}-0.5\right), & \Delta t\leq 0, \end{array}\;\;\;A_{\text{plus}}=A_{\text{sub}}. \end{array}$$

**Figure 2:**
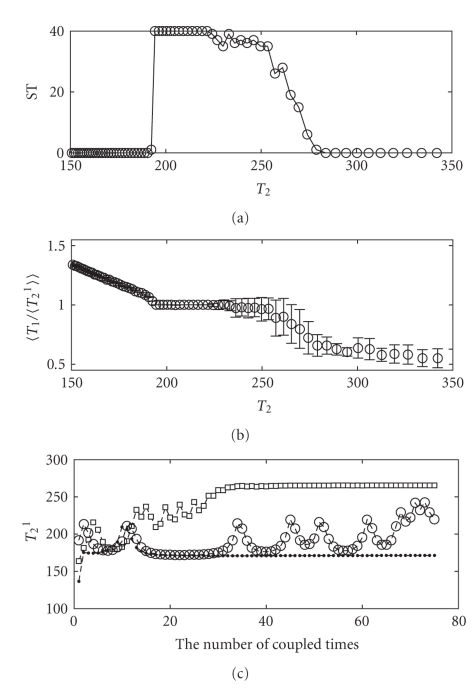
Synchronization results for dc-STDP rule carrying out 40 times. Figure 2(a) shows the number of synchronization times (ST) of fixed *T*_2_ varying from 150 milliseconds to 320 milliseconds. Synchronization window is from 194 to 221. The probabilistic synchronization window is from 222 to 289. Figure 2(b) presents the quality of synchronization against the ratios of uncoupled periods. In Figure 2(c), we fix *T*_2_ = 265 milliseconds which falls into the probabilistic synchronization window. There are three states of the coupled period of post-synaptic neuron *T*_2_^1^ when we carry out 40 stimulation times: (i) keeping the initial period (squares); (ii) oscillating (circles); (iii) synchronizing with the pre-synaptic neuron (dots).

Synchronization of pre- and post-synaptic neurons occurs when |*T*_1_ − 〈*T*_2_^1^〉| is limited in an acceptable range. We set the criteria of synchronization as |*T*_1_ − 〈*T*_2_^1^〉| < 1.5 milliseconds. Although there is some arbitrariness in setting the criteria of synchronization, there is no qualitative change in our results if the criteria change in two folds. Each simulation runs 20 seconds, average is taken in the final 4000 milliseconds. We have observed that simulations from different initial values of *V*_2_, *S* could result in different outputs, that is, the post-synaptic neuron sometimes synchronizes with the pre-synaptic one, sometimes keep its original period, or sometimes fires with an oscillating period (see Figure 2(c)). We therefore carry out 40 times of simulations, from randomly selected initial values, for every *T*_2_. The standard deviation of |*T*_1_ − 〈*T*_2_^1^〉|, indicating how precisely the neurons are synchronized, represents the quality of synchronization. Range of *T*_2_, in which post-synaptic neuron is successfully entrained by the pre-synaptic neuron, that is, |*T*_1_ − 〈*T*_2_^1^〉| < 1.5 in all 40 simulations, is defined as the synchronization window.

## 3. Results

### 3.1. Comparison of Synchronization Windows of Different Types of STDP

We investigate the width of synchronization window of various STDP curves, with the same set of parameters. The period of the pre-synaptic neuron is chosen to be 171 milliseconds, which falls into the range of theta waves. Several reasons make us choose such a long period. First, it has fairly wide synchronization windows which allow comparisons in a relative precise manner and can provide clearer information about synchronization windows of various STDP learning rules. Second, the slow theta waves always involve many neurons that fire synchronously [38, 39]. Also, theta waves have many interesting implications. For example, theta waves are normally absent in healthy awake adults, but appear during the state of meditation [40]. During emotional arousal and various types of rhythmic activities during sleep, neurons in the amygdala produce theta activity [41, 42]. And it is known that coherent theta activity (4–8 Hz) in amygdala-hippocampal circuits is deeply involved in fear memory [43].

With fixed period of the pre-synaptic neuron *T*_1_, we evaluate the coupled period of postsynaptic neuron *T*_2_^1^ when it is driven by the pre-synaptic neuron. The values of learning parameters used in c-STDP, dc-STDP, dc-aSTDP are *A*_plus_ = 9 nS, *A*_sub_ = 6 nS, *t*_plus_ = 100 milliseconds, *t*_sub_ = 200 milliseconds, *additionally τ*_0_ = 30 *milliseconds in c-STDP*, and in in-STDP are *A*_plus_ = *A*_sub_ = 8 nS, *t*_plus_ = 100 milliseconds, *t*_sub_ = 200 milliseconds. Our model and most values of parameters are from the model of Nowotny for c-STDP, except *A*_plus_, *t*_syn_, *V*_slope_, *g*_max_ are different [31]. Especially, *T*_1_ is fixed at 171 milliseconds in our simulations while *T*_2_ is set to constant value 300 milliseconds of Nowotny's work.

The window of synchronization (upper panel) and quality (middle panel) of dc-STDP are presented in Figure 2 as an example. We scan *T*_2_ from 150 milliseconds to 320 milliseconds. The upper panel shows the number of synchronization times in 40 simulations, for each *T*_2_. It is clear that, in certain range of *T*_2_, simulations from different initial values may have different results. Only when *T*_2_ falls into the segment from 194 to 221, the post-synaptic neuron can synchronize with the pre-synaptic neuron from any initial value. It is easily found that there are some *T*_2_ corresponding to the number of synchronization times between 1 and 39. In this situation, we present the three possible states of post-synaptic neuron's firing in Figure 2 (lower panel)—keeping the initial period (squares), oscillating (circles), and synchronizing with the pre-synaptic neuron (dots). Obviously, these states are independent of the synchronization criteria we set.

**Figure 3:**
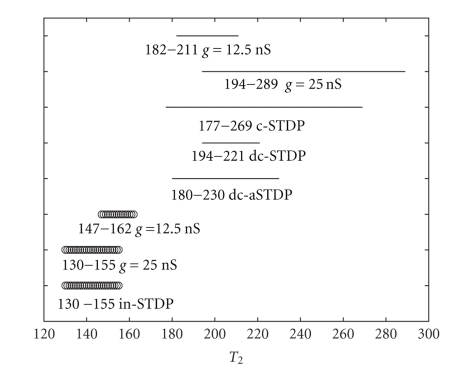
The synchronization window of different types of learning curves. The top five lines (points) are excitatory synapses and the bottom three lines (circles) are inhibitory synapses. See legends in the figure.

For the purpose of discussing the function role of STDP rules in synchronization, the synchronization windows of various type of STDP are plotted in Figure 3, in which the case of constant synaptic conductance is also included as a comparative tool. The same parameter values used in simulations ensure a fair comparison. In our simulation studies, the synapse strength is between 0 nS and 25 nS. We choose the maximal synaptic strength and the middle synaptic strength of STDP synapse as the synaptic strength of constant synapse in this study. Interestingly, because the synchronization windows for STDP rules are narrower than synchronization window for constant synapse *g* = 25 nS in Figure 3, these results, opposite to previous reports, indicate that all STDP rules do not enhance synchronization comparing with the constant synapse under the chosen parameters.

Several other points are worthy of detailed describing. First, increasing the excitatory constant synaptic connections from 12.5 nS to 25 nS, leads to a wider synchronization window. However, the wider window could not totally contain the smaller one. It extends toward larger *T*_2_, but loses a portion of smaller *T*_2_. The case of increasing inhibitory constant synaptic connections is alike. Second, the widest ranges of synchronization window are achieved by the excitatory constant connection *g* = 25 nS and c-STDP rules. However, the lower boundary of the synchronization window of c-STDP is much nearer to *T*_1_ than that of excitatory constant synapse. Thirdly, although c-STDP and dc-STDP are fitted from the same set of experimental data, c-STDP has a much wider synchronization window than dc-STDP.

We conclude that all STDPs do not give rise to enhanced synchronization and the window of dc-STDP is surprisingly narrow under the chosen parameters in Figure 3. Therefore, the questions about what bring about these results inspire us to study further. At the same time, Figure 2 shows a large part of probabilistic synchronization, whose range is a subset of the synchronization window of constant synaptic connection with *g* = 25 nS. We are interested in if the probabilistic synchronization could be enhanced into absolute synchronization by modulation of learning curves. These are the theme of the next section.

### 3.2. The Effect of Learning Parameters on Synchronization

In order to establish the functional role of STDP clearly, we consider if the learning parameters for each STDP rule have important effect on synchronization. In addition, synchrony-asynchrony transition plays important role in the brain. An increase in the degree of synchrony of a uniform input can cause transitions between memorized activity patterns in the order presented during learning. However, if synchronous input is at a low level, transitions cannot occur [44]. The synchrony-asynchrony transition have also been implemented in controlling winner-take-all competition [45], the next recalled time of associative memory [46] and the fine structure of cell assemblies [47].

In this section, we will discuss the flexibility of the synchronization window, by exploring regulation of width of the synchronization window, whose boundary indicates the synchrony-asynchrony transition. We take the modulation of learning parameters as the method to regulate the synchronization window. There are four parameters that determine a learning rule *A*_plus_, *A*_sub_, *t*_plus_, *t*_sub_. With three of them fixed and only one parameter changing, we could explore its influence on the width of the synchronization window. For example, *A*_plus_ increases from an adequately small value 1 nS to 20 nS with a step of 1 nS. We have also scanned values that are beyond 20 nS, but find that the effect of increasing *A*_plus_ is saturated around 20 nS. Further increasing *A*_plus_ brings no more effect. Other parameters are fixed as: *A*_sub_ = 6 nS, *t*_plus_ = 100 milliseconds, *t*_sub_ = 200 milliseconds, *T*_2_ = 233 milliseconds. In these conditions, we present the effect of *A*_plus_ on the location of the synchronization window with the dc-STDP rule. We carry out simulations 40 times, each from different initial values.

**Figure 4:**
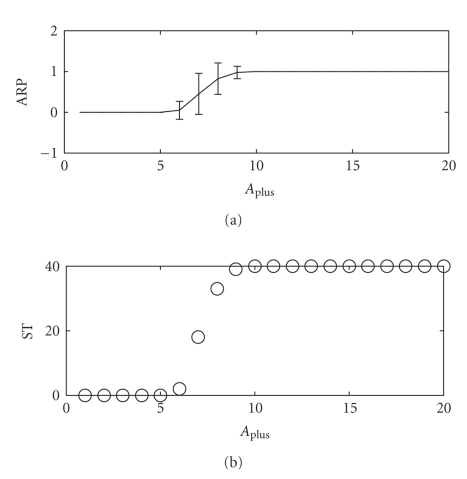
It shows that the regulation of *A*_plus_ to synchronization for the dc-STDP carrying out 40 times of stimulations. *A*_sub_ = 6 nS, *t*_plus_ = 100 milliseconds, *t*_sub_ = 200 milliseconds, *T*_2_ = 233 milliseconds. Top: the value of ARP = 〈*T*_2_ − 〈*T*_2_^1^〉〉/(*T*_2_ − *T*_1_) is 0 or 1 which, respectively, means post-synaptic neuron keeping initial period or achieving synchronization. Bottom: the number of synchronization times (ST) in 40 stimulations against *A*_plus_.

Figure 4(a) shows the value of ARP (average change of relative period ratio) = 〈*T*_2_ − 〈*T*_2_^1^〉〉/(*T*_2_ − *T*_1_) for different *A*_plus_. Some points have value 0 or 1, which means post-synaptic neuron keeping initial period or achieving synchronization with the pre-synaptic one, respectively. Some points have values other than 0 or 1. Figure 4(b) gives an explanation that these points correspond to probabilistic synchronizations with fixed *T*_2_ = 233.

We find that the absolute synchronization range is from 10 nS to 20 nS in Figure 4(b). According to the definition of synchronization window of *T*_2_, we can similarly define 10 nS to 20 nS as the synchronization window of *A*_plus_, with dc-STDP rule and other fixed parameters. The boundary of this synchronization window indicates where synchrony-asynchrony transition happens when changing *A*_plus_.

**Figure 5:**
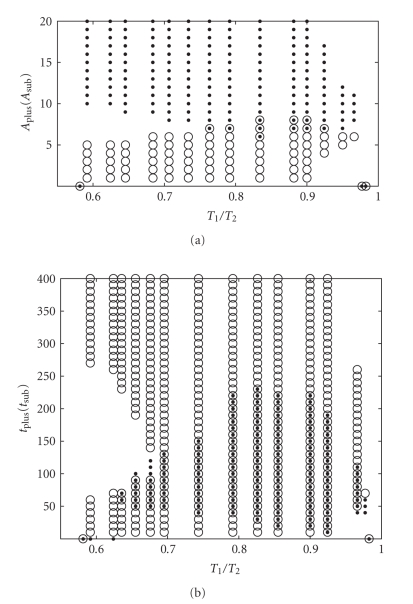
The range of learning parameters leading to synchronization for all 40 stimulations for c-STDP is presented. The points are the range of *A*_plus_ and *t*_plus_ which can make neuron synchronization. The circles are the range of *A*_sub_ and *t*_sub_. We choose some values of *T*_2_ to investigate the effect of *A*_plus_, *A*_sub_, *t*_plus_ and *t*_sub_ on global synchronization. *A*_plus_ and *A*_sub_ vary from 1 nS to 20 nS. Let *t*_plus_ and *t*_sub_ vary from 10 milliseconds to 400 milliseconds. There are some values of *T*_1_/*T*_2_ marked on the horizontal axis that cannot be entrained to achieve synchronization in Figures 5(a) and 5(b).

From the results of Figure 4, the reason why synchronization window of constant synapse is wider than that of STDP rules (Figure 3) may be explained by learning parameters. To figure out a global picture of the effect of *A*_plus_ on synchronization for c-STDP rule, we then determine the synchronization window of *A*_plus_ with different *T*_2_. *A*_plus_ increases from 1 nS to 20 nS with a step of 1 nS while other three parameters keep initial values: *A*_sub_ = 6 nS, *t*_plus_ = 100 milliseconds, *t*_sub_ = 200 milliseconds. We choose some typical values of *T*_2_ to character the global picture. The points in Figure 5(a) show the synchronization range of *A*_plus_ with *T*_1_ divided by the chosen values of *T*_2_. The lower boundaries, as well as those upper boundaries that are other than 20 nS, indicate the position of synchrony-asynchrony transitions. For example, when *T*_2_ is 177 milliseconds equivalent to *T*_1_/*T*_2_ = 0.966, *A*_plus_ outside of the points range from 8 nS to 11 nS cannot lead to synchronization between the two neurons. In addition, for those points marked on the horizontal axis, synchronization could not be established no matter what values *A*_plus_ take.

According to this global picture, the intersection of those synchronization ranges of *A*_plus_, which is from 10 nS to 11 nS, identifies the range of *A*_plus_ that would lead to the widest synchronization window which is from 177 to 289. This optimal synchronization window for c-STDP is wider than constant synapse *g* = 25 nS. Thus, the learning parameters strongly influence the role of STDP on synchronization.

Taking the same method as *A*_plus_, we study the effects of other three parameters on synchronization. Figure 5 presents the situation of *A*_sub_ varying from 1 nS to 20 nS with the step 1 nS (circles, Figure 5(a)), *t*_plus_ (points, Figure 5(b)) and *t*_sub_ (circles, Figure 5(b)) both varying from 10 milliseconds to 400 milliseconds with the step 10 milliseconds. When changing one parameter to explore how the range of synchrony evolves with *T*_2_, other three parameters keep their initial values as in Figure 3. Similarly, the global modulation picture of four learning parameters for other learning rules can be got. We only give the situation for c-STDP rule in Figure 5.

**Figure 6:**
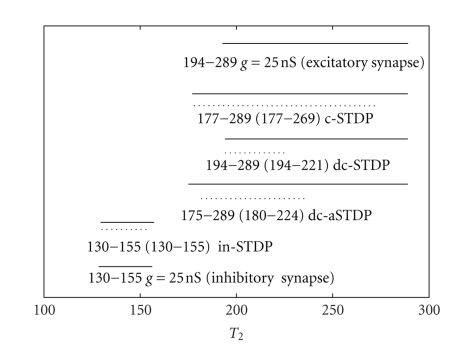
We present the optimal synchronization window for various learning rules compared with Figure 3. The top and the bottom lines of this figure are the synchronization windows of excitatory and inhibitory constant synapse, respectively. In addition, there are four pair lines in the middle panel for STDP rules. Each pair includes optimal synchronization window (top line) and previous synchronization window in Figure 3 (bottom line). The left range is optimal synchronization range and the range in round bracket is previous synchronization range.

The optimal synchronization windows for various learning rules are presented in Figure 6 comparing with previous synchronization windows obtained in Figure 3. The parameters used for optimal synchronization windows are presented in Table 1. The parameters are derived according to regulating one parameter while other three parameters keep initial values. Under the optimal parameters, the synchronization windows for STDP rules are not narrower than constant synapse *g* = 25 nS. Consequently, the reported important role of STDP in synchronization should be dependent on learning parameters.

For the first three STDP rules, the optimal synchronization windows are got by regulating *t*_sub_ while other three fixed learning parameters keep initial values. For the last STDP rule, the initial values are the optimal parameters.

### 3.3. The Synchronization Mechanism

It is important to understand the properties which neural synchronization depends on. We take into account this problem from two aspects.

On the one hand, synchronization correlates with the chosen STDP rules. For excitatory synapse, we find that the optimal synchronization windows for c-STDP rule and dc-aSTDP rule are almost equal, and are wider than constant synapses. However, comparing with these two rules, the optimal synchronization window for dc-STDP rule is much narrower. For inhibitory STDP, the synchronization window is the same as that for constant synapse under connection strength *g* = 25 nS.

Accordingly, what makes the optimal synchronization windows for various STDP rules different deserves an explanation. The stationary synaptic conductance is a necessary condition for stationary synchronized state [31]. We find that the mechanisms of synchronization caused by c-STDP (or dc-aSTDP) and dc-STDP (or in-STDP) are different in our model.

**Figure 7:**
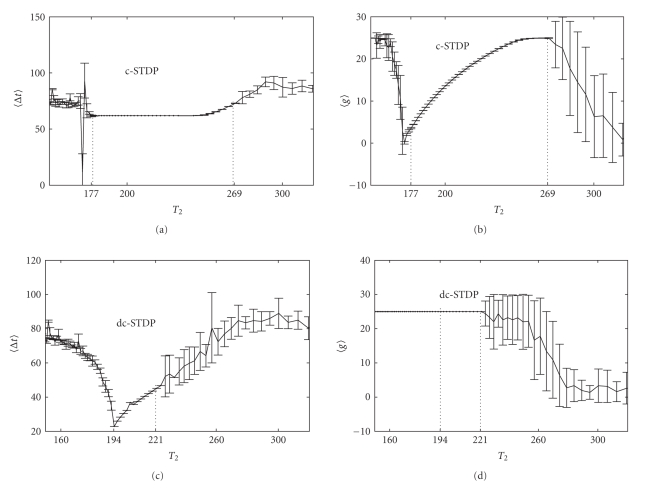
Four pictures are obtained from the same values of parameters and model of Figure 3. The front four pictures take the function of tanh to limit synaptic strength for c-STDP and dc-STDP rules. Figures 7(a)  and 7(c)  show the average spike time interval of postneuron's and preneuron's spike time for c-STDP and dc-STDP over some time after a period time of coupling, respectively. Figures 7(b)  and 7(d)  present the average synaptic strength for c-STDP and dc-STDP rule, respectively. Each subplot has two dash lines what indicate the boundary of synchronization window.

Figures 7(a) and 7(b)  show the average Δ*t* = *t*_postspike_ − *t*_prespike_, and synaptic strength after an episode of coupling time for c-STDP rule. Parameters in Figure 7 are same as those in Figure 3. There are two types of behavior for Δ*t* when synchronization occurs (Figure 7(a)). In a section of constant Δ*t*, the synaptic strength does not achieve the maximal value. Apparently, in this situation, postsynaptic neuron achieves synchronization with the pre-synaptic neuron depending on the balance between potentiation and depression of synaptic conductance. In the rest part of synchronization window, the synaptic strength achieves the maximal value (Figure 7(b)). It indicates that for larger *T*_2_, postsynaptic neuron achieves synchronization depending on the effect of maximal synaptic conductance. For dc-aSTDP rule, the synchronization mechanisms are similar to c-STDP rule. When the post-synaptic neuron synchronizes with the pre-synaptic neuron under small *T*_2_, the change of synaptic potentiation and depression cancel each other. However, for the small portion of synchronization window at the right side, synaptic conductance gets the maximum at the stationary synchronized state.

For dc-STDP and in-STDP rules, the synchronization mechanisms may be different with the above two STDP rules. Because the potentiation and depression of synaptic conductance cancel each other, Δ*t* must be a fixed value for the selected STDP rule. However, for the dc-STDP rule (Figures 7(c) and 7(d)), Δ*t* keeps varying which means that the potentiation and depression of synaptic conductance do not cancel each other at the synchronization state. But, it is easily found that the synaptic conductance is at the stationary maximum for dc-STDP rule. Thus, postsynaptic neuron achieves synchronization completely depending on the effect of the maximal synaptic conductance for dc-STDP rule. The state of in-STDP rule is similar to dc-STDP rule.

As a result, neural synchronization mechanism can be different for various STDP rules. For the few *T*_2_ at the right side of the synchronization window, the synaptic conductance achieves the maximum with c-STDP and dc-aSTDP rules. This result is obvious. Because the frequency mismatch is larger, the synapse needs to be stronger to entrain the post-synaptic neuron. But for most part of synchronization window at the left, c-STDP and dc-aSTDP rules rely on the balance of potentiation and depression. Instead of balancing out potentiation and depression during one cycle, dc-STDP and in-STDP rules depend on synaptic strength achieving its maximum. It is important to understand why c-STDP and dc-aSTDP rely on the balance of potentiation and depression while dc-STDP and in-STDP do not.

**Figure 8:**
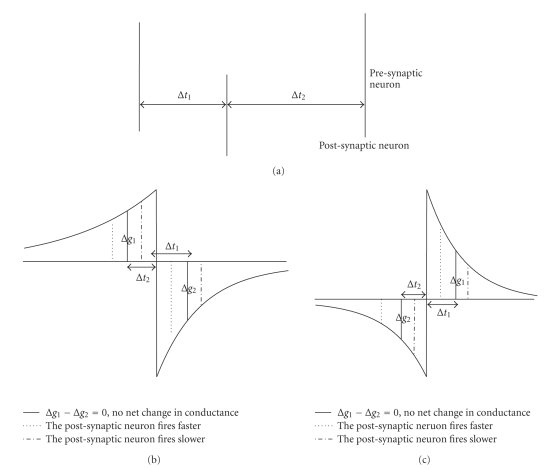
Different synchronization mechanisms for different STDP rules. (a) Shows the situation of Δ*t*_1_ − Δ*t*_2_ = *T*_1_ = *T*_2_, where Δ*g*_1_ − Δ*g*_2_ = 0. The solid lines in (b) and (c) are same as (a). (b) and (c) present the change of synaptic strength of dc-aSTDP and dc-STDP rules, respectively.

Nowotny et al. have introduced the mechanisms behind the enhancement of neural synchronization by c-STDP rule which rely on the balance of potentiation and depression. The synapse strength remains stable regardless of postsynaptic neuron firing later or earlier attributed to the specific shape of c-STDP curve. The situation of c-STDP is similar to dc-aSTDP. We adopt the similar analysis method [31] here for the dc-aSTDP and dc-STDP. The time lags are recorded as Δ*t*_1_ and Δ*t*_2_, where Δ*t*_1_ − Δ*t*_2_ = *T*_1_ = *T*_2_^1^ and Δ*g*_1_ − Δ*g*_2_ = 0 (Figure 8) at this state. If post-synaptic neuron fires faster, Δ*t*_1_ becomes smaller. Synaptic strength will be depressed, due to Δ*g*_1_ − Δ*g*_2_ < 0 for dc-aSTDP rule, so that the post-synaptic neuron is less excited and goes back into the synchronized state (Figure 8(b)). The other direction can be analyzed in the same way for dc-aSTDP. But for dc-STDP, when post-synaptic neuron fires faster, synaptic strength will be increased due to Δ*g*_1_ − Δ*g*_2_ > 0 in this case. The post-synaptic neuron is more excited and cannot go back into the synchronized state (Figure 8(c)). The opposite direction is the same case for dc-STDP and cannot go back into the synchronized state. Therefore, the synchronization mechanisms between these two rules are different.

For in-STDP rule, we can easily find that Δ*g*_1_ − Δ*g*_2_ is always positive, where Δ*g*_1_ − Δ*g*_2_ = *A*_plus_ ∗ (exp(−*x*/*t*_plus_)−0.5) − *A*_sub_ ∗ (exp(*x*−171/*t*_sub_) −0.5) and values of parameters are the same as Figure 3. It means that the potentiation and depression of synaptic conductance during one period cannot achieve balance. The synaptic strength must achieve the maximum resulting from Δ*g*_1_ − Δ*g*_2_ > 0.

On the other hand, learning parameters also play important role in neural synchronization. We try to explain the role of a learning parameter by considering how it influences the synapse conductance, which is a major factor for synchronizing neurons with a given mismatch of intrinsic frequencies.

It is obvious that if synaptic conductance becomes stronger, it can make larger *T*_2_ to achieve the same period with pre-synaptic neuron for various STDP rules. Thus, with other three learning parameters fixed, larger *A*_plus_ values, which are corresponding to the stronger stable synaptic strength for c-STDP rule, can cause larger *T*_2_ synchronization. Similarly, smaller *A*_sub_ values will give rise to larger *T*_2_ synchronization for c-STDP (Figure 5(a)). Moreover, from Figure 5(b), moderate *t*_plus_ values can also make larger *T*_2_ synchronized to *T*_1_ because these values bring about stronger synaptic strength. We can prove this perspective by simple calculus reasoning. Based on the expression of c-STDP rule in Section 2, let *t*_plus_ be variable and let other parameters keep constant. Δ*g*_raw_ is viewed as the function of variable *t*_plus_. By calculating the derivative of Δ*g*_raw_, we can find that Δ*g*_raw_ is a first increasing and then decreasing function when *t*_plus_ increases gradually. Thus, medial values of *t*_plus_ can result in stronger synaptic strength. By the same reasoning for *t*_sub_, we can conclude that smaller or larger *t*_sub_ can make larger *T*_2_ synchronization for c-STDP (Figure 5(b)).

The effects of learning parameters on synchronization about other learning rules are similar to the c-STDP rule for larger *T*_2_. However, smaller *T*_2_ values leading to synchrony are only related to c-STDP and dc-aSTDP, because they rely on the balance of depression and potentiation which could lead to an appropriate low stable synaptic strength. Therefore, only proper learning parameters got by regulating the effect of learning parameters on synchronization are required for smaller *T*_2_ achieved synchronization.

Finally, we conclude why the widest synchronization windows for some STDP rules are different. From the above statements, we find that two aspects affect synchronization. One is the maximum synaptic strength which can make larger *T*_2_ synchronize. The other is the balance of depression and potentiation which can make smaller and moderate *T*_2_ synchronize. For c-STDP and dc-aSTDP, they can achieve the widest synchronization windows through modulating the two aspects. However, dc- STDP and in- STDP rules, due to their specific shape, can only make use of the first one. This implies that the widest synchronization windows for dc-STDP and in-STDP rules cannot exceed the synchronization windows for the maximum constant connection strength under the same model respectively. Therefore, the optimal synchronization windows for c-STDP and dc-aSTDP are wider than those for dc-STDP and in-STDP.

## 4. Conclusion

STDP plays important functional role in neural synchronization. The mechanism of STDP in neuronal synchronization is still not completely clear. Inspired by previous experiments and theoretical researches, we study the important aspects of STDP-induced synchronization in this paper, such as the role of various STDP rule in synchronization, the widest synchronization window through regulating learning parameters, and synchronization mechanism.

In order to explore the functional role of STDP in synchronization, we compare synchronization windows of different types of STDP rules with that of constant synapse under the same model parameters. For the given parameters, not all synchronization windows are enhanced by STDP rules.

Synchronized responses have a stronger influence on cells at subsequent processing stages than nonsynchronized responses [48–50]. And the enhanced precise synchronization is important in improving a rapid and reliable transmission of information about sensory changes [2, 51]. Recent researches have reported various methods to enhance synchronization, such as, selective attention [52] and time delay [53]. Here, we present the effect of modulation of learning parameters on synchronization and optimal synchronization window which are not narrower than constant synapse. The optimal synchronization windows by c-STDP and dc-aSTDP rules are much wider than constant synapse. It indicates that the function role of STDP rule in synchronization depends on the learning parameters.

The synchronization mechanism is also described here. Different shapes of STDP rule can cause different optimal synchronization windows. The optimal synchronization windows of c-STDP and dc-aSTDP are wider than that of dc-STDP for excitatory synapse. For c-STDP rule and dc-aSTDP rule, a stationary synchronized state completely depends on the balance between potentiation and depression or the maximal synaptic conductance. However, for dc-STDP and in-STDP rule, the stable synchronized state depends on the maximal synaptic conductance under the self-limitation of synaptic strength. If we change the type of bound of synaptic strength for dc-STDP rule from self-limitation to artificial bounds, we find that the synchronization mechanism does not change. In a word, on one hand, the synchronization range of dc-aSTDP and c-STDP can achieve the optimal synchronization window of dc-STDP, depending on the maximal synaptic conductance. On the other hand, dc-aSTDP and c-STDP can extend the synchronization windows to include smaller *T*_2_ by the balance between potentiation and depression.

The firing pattern of neurons is regular in this paper. Many neurons in brain areas present regular firing. For example, neurons in cat area 17 can be grouped in 4 different electrophysiological cell classes, including regular spiking [54]. And spontaneous, regular action potentials were observed both with cell-attached patch recordings as well as with whole cell current-clamp recordings for cholinergic neurons in the parabigeminal nucleus of the rat midbrain [55]. Neuronal synchronization properties with regular firing neurons have been studied. For example, whether pyramidal neurons in different cortical layers exhibit similar tendencies to synchronize is studied [56]. Based on this point and the functional role of STDP in synchronization, we explore the synchronization windows of various STDP rules from the view of neurons' regular firing.

STDP-mediated synchronization is a remarkably robust phenomenon against strong noise [30, 31]. Although our simulation is not under the noise environment, our results may represent some predictions for STDP-mediated synchronization in noisy environment. In addition, from Figure 2(a), we can clearly see that some synchronization number is between 1 and 39. We estimate that it may be related to phase.

Our results present that the range of *T*_2_ values leading to synchrony increases strongly if the constant synaptic connection is increased from 12.5 nS to 25 nS. Nowotny et al. find that the extent of synchrony does not change considerably by doubling the synaptic conductance [31]. Their result is not conflict with our result. There are three parameters that are different, *V*_slope_, *t*_syn_, and *g*_max_. Furthermore, *T*_1_ is fixed at 171 milliseconds in our simulation while *T*_2_ is fixed at 300 milliseconds in theirs. When *T*_2_ is fixed, the range of *T*_1_ values leading to synchrony is limited from 0 to *T*_2_ no matter how the strength of constant connection changes. However, when *T*_1_ is fixed, the range of *T*_2_ values leading to synchrony can vary from *T*_1_ to very large value due to the increase of the constant connection. At the same time, if we adopt the same parameters with Nowotny's paper, the similar result can be got. Furthermore, when constant synaptic connection is 0 nS, it is clear that two neurons with different initial periods cannot synchronize. This situation means that the length of synchronization window is 0. Along with the increase of strength of constant connection, some *T*_2_ must cause the synchronization. Thus, it is easily found that synchronization window must become wider by increasing the constant connection to some degree.

We mainly discuss synchronization for different STDP rules in this paper. The question of how the time windows of various STDP rules are biophysically regulated remains relatively unexplored. There are some experiments using neuromodulators to study the time window for STDP [57]. We are interested in building molecular kinetic equations for STDP to explain our results.

It has been proposed that conscious perception depends on the transient synchronization of widely distributed neural assemblies [58]. And long-distance synchronization plays a role in triggering the cognitive processes associated with conscious awareness [59]. The changed learning parameters by neuromodulators may influence the cognitive processes. In addition, some diseases and the function of brain are related with synchronization mentioned above, especially in theta (4–8 Hz) rhythm synchronization during fear memory retrieval [43] which is consistent with what we considered here. Therefore, our work may advance understanding of synchronization to some extent. And we expect that our simulation results will provide some help for related diseases treatment.

## Figures and Tables

**Table 1:** The parameters for optimal synchronization windows.

STDP rule	*A*_plus_ (nS)	*A*_sub_ (nS)	*t*_plus_ (ms)	*t*_sub_ (ms)
c-STDP	9	6	100	270
dc-STDP	9	6	100	50
dc-aSTDP	9	6	100	350
in-STDP	8	8	100	200
